# Notes and News

**Published:** 1895-02-16

**Authors:** 


### Notes and News.

Collected and Communicated.

HOSPITAL MEETING.

Great Northern Central Hospital.—Annual meeting at the Hospital, on February 22nd, at 5 p.m.

The new lying-in hospital in New York is now open. It contains five wards with thirty beds, an operating theatre, and all modern arrangements. The old building is being retained as an emergency station. At Portland, Oregon, a new hospital for women has been opened.

The use of arsenic as a beautifying agent has become so large in America that the Board of Health is petitioning Congress to restrict the sale. It is said that very much larger doses are made up and sold to improve the complexion than purchasers are themselves aware of, and that health is thus permanently injured.

A special meeting of the council of the Charity Organisation Society was held on Monday at the Hotel Victoria, Northumberland Avenue, when Colonel Montefiore, secretary of the Medical and Convalescent Sub-Committee, read a paper on the need of a Central Hospital Board in London, with special reference to the promotion of uniformity in the system of admission to hospitals. Mr. Bousfield, chairman of the council, presided.

The Lord Mayor presided, last week, at the Mansion House, over a meeting of the newly-elected council of the Metropolitan Hospital Sunday Fund. The proceedings were formal. Resolutions were passed appointing the general purposes committee, the committee of distribution, and the surgical appliances committee, the latter consisting of any two members of the council, acting with the secretary. It was resolved to ask Sir E. H. Currie and Mr. R. Biddulph Martin, M.P., again to serve as hon. secretaries, and Mr. Custance was reappointed the secretary, a position which he stated he had held from the establishment of the fund. Messrs. W. H. Pannell and Co. were appointed hon. auditors.

We regret to announce the death of Mr. William Tresidder, secretary of the Surgical Aid Society for the past 32 years, who died on the 11th inst. He will be greatly missed by the governors, as the society owes much of its prosperity to Mr. Tresidder's devotion to its interests.

We learn that there is an opening in Cape Colony for a thoroughly qualified medical officer of health. The salary is £1,000 a year. Applications (supported by testimonials and stating the candidate's age, date when he would be prepared to enter upon his duties, &c.), will be received by the Agent-General, 112, Victoria Street, S.W., and by the Under-Colonial Secretary at Cape Town up to noon on March 15th next.

It is stated that Sydney has set the example of attempting to suppress by law the habit of spitting, which is now held by many medical men to be largely instrumental in the spread of consumption In America a society for the prevention of tuberculosis recently recommended that a regulation should be passed, making it a misdemeanour to expectorate in any public conveyance, but the suggestion was not carried out. Any person convicted of spitting upon the floor of a public building or in the street in Sydney is to be fined £1. Of course, the difficulty will be to find prosecutors, and, again, so rooted is the habit of spitting amongst members of the masculine sex especially, that many well brought up and educated gentlemen highly resent the idea of this restriction of the liberty of the subject. The state of the smoking carriages in our railway carriages, especially the third-class compartments, is a disgrace to civilisation, and the question has therefore to be faced, whether smoking is the sole excuse for the habit of spitting in men, or if it is a necessity to health? If it is necessary, the restraint which women place upon themselves in not yielding to this habit must surely be injurious to them.

In spite of "the severe weather," the public meeting on behalf of St. Thomas's Hospital, held in the Egyptian Hall of the Mansion House, on Wednesday, was very well attended. In the unavoidable absence of the Lord Mayor, through illness, the chair was taken by Sir Stuart Knill, who was supported by H.R.H. the Duke of Connaught, the President of the hospital. The Archbishop of Canterbury was unable to be present, as was also the Bishop of Rochester (on account of sudden illness): The Duke of Connaught made an earnest appeal to those present to come forward and help the hospital in its present hour of need, and speeches followed by Sir William MacCormac, the Rev. Norman Hall, the Rev. D. MacEwen, D.D., and others. A letter was read from Miss Florence Nightingale, whose warm interest in St. Thomas's Hospital is so well known, which elicited much applause. During the course of the meeting Mr. Wainwright, the treasurer, announced that H.M. the Queen had sent a contribution of £100 towards the fund, and further donations of 100 guineas and £100 from the Duke of Devonshire and Miss Florence Nightingale respectively. A full report of the meeting will be given next week.

